# Intrauterine devices influence prostaglandin secretion by equine uterus: in vitro and in vivo studies

**DOI:** 10.1186/s12917-024-03889-0

**Published:** 2024-02-03

**Authors:** Katarzyna Karolina Piotrowska-Tomala, Agnieszka Walentyna Jonczyk, Anna Szóstek-Mioduchowska, Takuo Hojo, Ewelina Żebrowska, Terttu Katila, Graca Ferreira-Dias, Dariusz Jan Skarzynski

**Affiliations:** 1 Department of Reproductive Immunology and Pathology, Institute of Animal Reproduction and Food Research, Polish Academy of Sciences, Tuwima 10 St., 10-747 Olsztyn, Poland; 2 Kyushu Okinawa Agricultural Research Center, NARO, 2421 Suya, Koshi, Kumamoto 861-1192 Japan; 3 Department of Production Animal Medicine, Faculty of Veterinary Medicine, University of Helsinki, Paroninkuja 20, 04920 Saarentaus, Finland; 4 Faculty of Veterinary Medicine, CIISA-Centre for Interdisciplinary Research in Animal Health, University of Lisbon, 1300-477 Lisbon, Portugal

**Keywords:** Prostaglandin synthases, Prostaglandin F_2α_, Prostaglandin E_2_, Endometrial cells, Horse

## Abstract

**Background:**

Intrauterine devices (IUD) are used in the veterinary practice as the non-pharmacological method of oestrus suppression in mares. When placed in the uterus, IUD create a physical contact with the endometrium that mimics the presence of an equine embryo. However, the mechanism of their action has not been fully elucidated. The objective of the present study was to examine the effect of mechanical stimulation of IUD on mare`s endometrium in both in vitro and in vivo study. For this purpose, we demonstrated the effect of IUD on prostaglandin (PG) F_2α_ and PGE_2_ secretion, and mRNA transcription of genes involved in PG synthesis pathway in equine endometrial cells in vitro. In the in vivo study, we aimed to compare short-term effect of IUD inserted on day 0 (oestrus) with day 5–6 post-ovulation (the specific time when embryo reaches uterus after fertilization) on PG secretion from equine endometrium. To determine the long-term effect on *PG synthase* mRNA transcription, a single endometrial biopsy was taken only once within each group of mares at certain time points of the estrous cycle from mares placement with IUD on days 0 or 5–6 post-ovualtion.

**Results:**

We showed for the first time that the incubation of the endometrial cells with the presence of IUD altered the pattern of *PG synthase* mRNA transcription in equine epithelial and stromal endometrial cells. In vivo, in mares placement with IUD on day 0, PGE_2_ concentrations in blood plasma were upregulated between 1 and 6, and at 10 h after the IUD insertion, compared with the control mares (*P* < 0.05). Moreover, the decrease of *PTGFS* mRNA transcription on day 16- 18, associated with an elevation in *PTGES* mRNA transcription on day 20 -21 of the estrous cycle in endometrial biopsies collected from mares placement with IUD on days 5–6 suggest an antiluteolytic action of IUD during the estrous cycle.

**Conclusion:**

We conclude that the application of IUD may mimic the equine conceptus presence through the physical contact with the endometrium altering *PG synthase* transcription, and act as a potent modulator of endometrial PG secretion both in vitro and in vivo.

## Background

Embryo-maternal communication and possible embryo-derived signal involved in the process of maternal recognition of pregnancy (MRP) are not fully known, despite many years of research on this topic [1–4]. The embryo reaches the uterus between days 5 and 6 after fertilization [5]. Until day 16, the equine conceptus is unattached within the uterus, and myometrial contractions move the embryo actively throughout the uterine lumen [6]. Then the embryo becomes static in the uterine horn, as a result of its enlargement and increased uterine tone [7]. It is well known that in the non-pregnant mare, prostaglandin (PG) F_2α_ terminates the corpus luteum (CL) lifespan [8]. However, intrauterine presence and migration of the embryo during early gestation are involved in the inhibition of PGF_2α_ release [9–11] leading to continued secretion of progesterone (P_4_) by the CL, which is a crucial mechanism in the process called MRP [12]. Restricted embryonic mobility may lead to luteolysis, P_4_ reduction, and consequently to early pregnancy loss [11]. The inhibitory effect of the equine embryo on endometrial PGF_2α_ secretion has been observed in vivo [3, 13]. In vitro, Berglund et al. [14] and Watson and Sertich [15] demonstrated that incubation of endometrial explants with embryonic vesicles decreased PGF_2α_ released by the endometrium.

Intrauterine devices (IUD), such as a small sphere made of glass or other material have been used in veterinary practice as the non-pharmacological method of oestrus suppression in mares [16]. Nie et al. [16] and Rivera del Alamo et al. [17] speculated that IUD mimics an embryo or acts via an induced chronic uterine inflammation, that decreases the release of uterine PGF_2α_ amount resulting in inhibition of luteolysis. Previous studies have shown that inserting a plastic ball into the mare’s uterus during early dioestrus prolongs the luteal lifespan by suppressing prostaglandin-endoperoxide synthases 2 (PTGS2) which leads to the inhibition of PGF_2α_ release suggesting that mechanical force or contact on the endometrium by IUD blocks luteolysis [17, 18]. The presence of plastic spheres in the uterus is restricted to the uterine body and horn base area [10]. The physical contact with or the pressure on the endometrial wall may mediate the signal of the presence of IUD to the endometrium, which will then respond similarly as in MRP [17, 18].

A recent study showed Annexin 1 (AnxA1) protein upregulation in the endometrial secretions of mares with IUD [19]. This was interpreted to indicate inflammation caused by the IUD, because AnxA1 has potent anti-inflammatory and pro-inflammatory activities. Annexin 1 has been shown to inhibit phospholipase 2, and the authors speculated that it contributes to the subsequent inhibition of PTGS2 and PGF_2α_ release [19]. However, AnxA1 plays a role also in blastocyst attachment by affecting human endometrial epithelium. It induces expression of proteins controlling paracellular flux and structure and adhesiveness of the human uterine wall [20]. Therefore, increased AnxA1 levels may also reflect mechanical interaction between the IUD and endometrial epithelium. In addition, the study of Rivera del Alamo et al. [19] showed that the presence of IUD changes the protein composition of endometrial secretions. However, the knowledge about the mechanism of IUD action on the secretory function of the equine uterus, especially on the changes of endometrial cells’ secretory function is still insufficient.

The aim of this study was to gain new insights into the mechanisms by which IUD modulate PG secretion in equine endometrium. We hypothesized that IUD that mimics the presence of an embryo in the uterus affecting PG production by its physical contact and mechanical stimulation of endometrial cells. For this purpose, we demonstrated the effect of IUD on PG secretion and mRNA transcription of genes involved in PG synthesis pathway in equine endometrial cells in vitro. Moreover, we assumed that the endometrial response to IUD presence may depend on the day of its application into the uterus. Therefore, we compared the short-term or long-term effect of an IUD inserted on day 0 (oestrus) with day 5–6 post-ovulation (the specific time when an embryo reaches uterus after fertilization) on (i) PG secretion from equine endometrium, and (ii) mRNA *PG synthase* transcription in vivo. In in vitro studies, inflammation is not present, and therefore its possible effect on PG release is excluded. Furthermore, the effect of frequency of biopsies on mRNA *PG synthase* transcription changes was included in the preliminary in vivo study.

## Results

### Experiment 1. The time-dependent effect of IUD on PG in endometrial cells in vitro

**Fig. 1:**
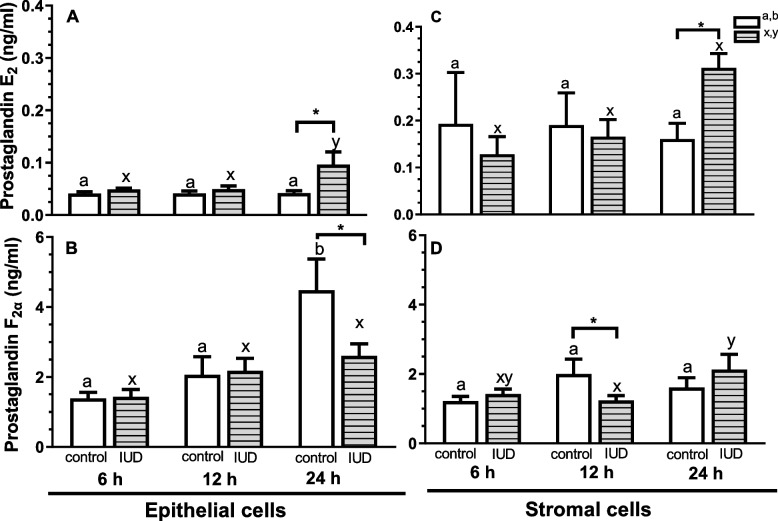
Effects of an intrauterine device (IUD) on prostaglandin E_2_ (**A**, **C**) and prostaglandin F_2α_ (**B**, **D**) secretion by equine epithelial (**A**, **B**) and stromal (**C**, **D**) cells, collected during the early luteal phase, after 6, 12, and 24 h of incubation with the presence of IUD. Asterisks indicate significant differences (^*^P < 0.05; ^**^P < 0.01) from the respective control. Superscript letters indicate significant differences between the control group (control ^a,b^) across the time points or between IUD mares (IUD ^x,y^) across the time points

In the epithelial cells incubated in the presence of IUD, concentration of PGE_2_ increased at 24 h, compared with the control group (the epithelial cells incubated without IUD) (*P* < 0.05; Fig. 1A), with no difference observed at 6 and 12 h (*P* > 0.05; Fig. 1A). While concentration of PGF_2α_ decreased when the cells were incubated in the presence of IUD for 24 h (*P* < 0.05; Fig. 1B). No difference in PGF_2α_ concentration was found in the epithelial cells at 6 and 12 h of incubation in the presence of IUD (*P* > 0.05; Fig. 1B). In the epithelial cells incubated in the presence of IUD, concentrations of PGE_2_ increased at 24 h, compared with IUD group at 6 and 12 h (*P* < 0.05; Fig. 1A). While concentrations of PGF_2α_ increased in the control group at 24 h, compared with its concentrations in control groups at 6 and 12 h (*P* < 0.05; Fig. 1B).

At 24 h, concentration of PGE_2_ increased in the stromal cells incubated in the presence of IUD, compared with the control group (the stromal cells incubated without IUD) (*P* < 0.05; Fig. 1C), with no difference in its concentration observed at 6 and 12 h (*P* > 0.05; Fig. 1C). Whereas concentration of PGF_2α_ decreased in the stromal cells at 12 h (*P* < 0.05; Fig. 1D), with no difference found at 6 and 24 h of incubation in the presence of IUD (*P* > 0.05; Fig. 1D). At 12 h, concentrations of PGF_2α_ decreased in the stromal cells incubated with IUD, compared with IUD group at 24 h (*P* < 0.05; Fig. 1D).

**Fig. 2:**
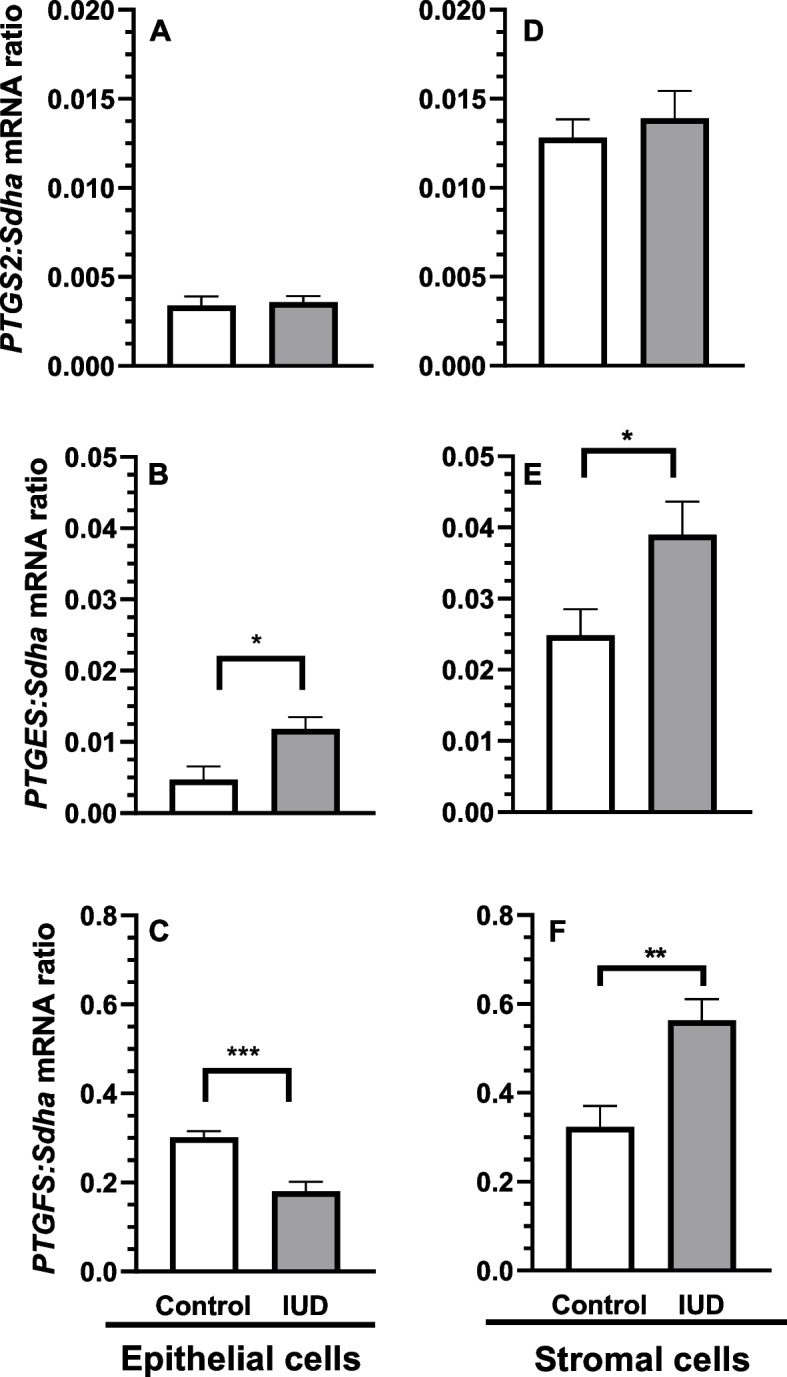
Effects of the intrauterine device (IUD) on transcripts of prostaglandin-endoperoxide synthase-2 (*PTGS2*) (**A**, **D**), prostaglandin E_2_ synthases (*PTGES*) (**B**, **E**) and prostaglandin F_2α_ synthases (*PTGFS*) (**C**, **F**) in equine epithelial (**A**, **B**, **C**) and stromal (**D**, **E**, **F**) cells, collected during the early luteal phase, after 24 h of incubation with the presence of IUD. Succinate dehydrogenase A (Sdha) – reference gene. Asterisks indicate significant differences (^*^P < 0.05; ^**^P < 0.01; ^***^P < 0.001) from the respective control

In the epithelial cells, incubation in the presence of IUD for 24 h enhanced *PTGES* mRNA transcription compared with the control group (the epithelial cells incubated without IUD) (*P* < 0.05; Fig. 2B), while *PTGFS* mRNA transcription decreased (*P* < 0.001; Fig. 2C). In the stromal cells incubated in the presence of IUD for 24 h *PTGES* and *PTGFS* mRNA transcription increased, in contrast to the control group (the stromal cells incubated without IUD) (*P* < 0.05 and *P* < 0.01, respectively; Fig. 2E and F). Regarding *PTGS2*, no difference was observed in its transcription in the epithelial and stromal cells (*P* > 0.05; Fig. 2A and D).

### Experiment 2. The short-term effect of the IUD inserted on day 0 versus days 5–6 of the estrous cycle on blood hormonal profile in mare in vivo

**Fig. 3:**
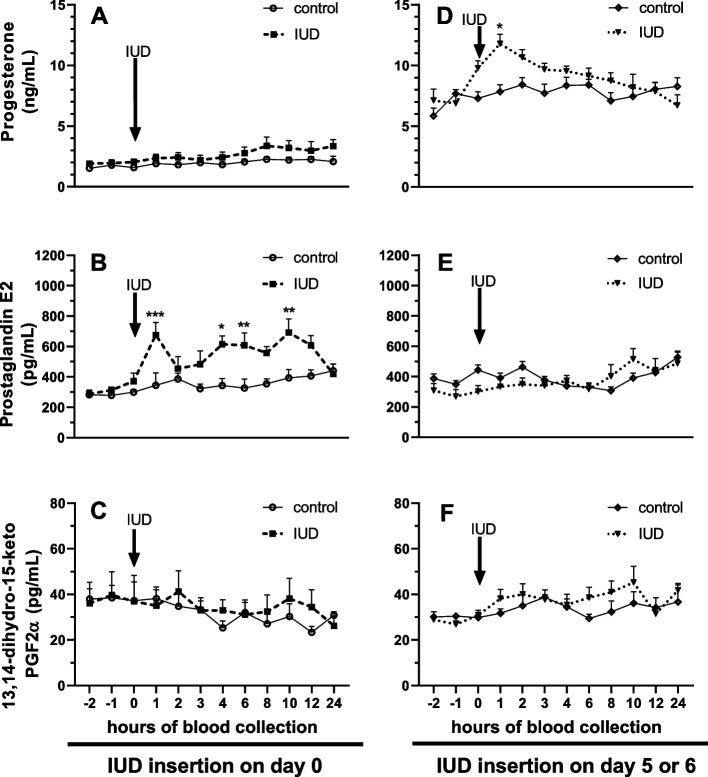
The short-term effect of an intrauterine device (IUD) inserted on day 0 (**A**,**B**, **C**) and IUD inserted on days 5–6 post-ovulation (**D**, **E**, **F**) on concentrations of progesterone (P_4_) (**A**, **D**), prostaglandin E_2_ (**B**, **E**), and 13,14-dihydro-15-keto PGF_2α_ (**C**, **F**) in the blood plasma of mares. Asterisks indicate significant differences (^*^P < 0.05) between IUD mares and control mares (without IUD) at the same time point (hour). Arrow indicates time point of IUD insertion

The endometrial response as PG release is different depending on the day of the estrous cycle of IUD insertion. In the mares placement with IUD on day 0 of the estrous cycle (D0_IUD), PGE_2_ concentrations in blood plasma were upregulated at 1, 4, 6, and 10 h after the IUD insertion, compared with the control mares (without IUD; D0_IUD) (*P* < 0.05; Fig. 3B). While no difference was observed in P_4_ and PGFM concentrations in collected blood plasma, compared with D0_CON (*P* > 0.05; Fig. 3A and C). In the D5_IUD, P_4_ concentrations in blood plasma increased temporarily in IUD mares 1 h after IUD insertion compared with the D5_CON (*P* < 0.05; Fig. 3D), while no difference was observed in PGE_2_ and PGFM concentrations in collected blood plasma, compared with the D5_CON (*P* > 0.05; Fig. 3E and F).

**Fig. 4:**
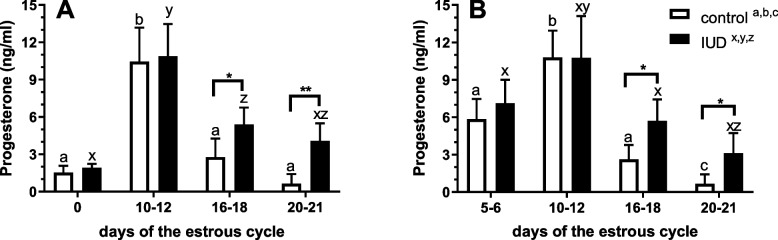
The effect of an intrauterine device (IUD) inserted on day 0 (**A**) versus IUD inserted on days 5–6 post-ovulation (**B**) on progesterone (P_4_) concentration in the blood plasma collected within the time intervals during the estrous cycle: day 0 and day 5 or 6 respectively, and days 10—12, 16- 18, 20—21 from IUD mares compared with control mares (without IUD) with blood samples collected on the same days of the estrous cycle. Asterisks indicate significant differences (^*^P < 0.05) between IUD mares and control mares (without IUD) at the same time point. Superscript letters indicate significant differences between the control group (control ^a,b,c^) across the time points (days of the estrous cycle) or between IUD mares (IUD.^x,y,z^) across the time points (days of the estrous cycle)

Progesterone concentrations were higher in blood samples collected from mares in both IUD groups (D0_IUD or D5_IUD) on days 16 -18 and 20- 21, respectively (*P* < 0.05; Fig. 4A and B), compared with D0_CON or D5_CON at the specific time points, confirming the effect of IUD on luteal persistence in mares. Progesterone concentrations were higher in blood samples collected from D0_CON or D0_IUD mares on day 10- 12 compared with days 0, 16 -18, and 20—21 (*P* < 0.001; Fig. 4A). In blood samples collected from D5_CON and D5_IUD mares, P_4_ concentrations were higher on day 10 -12 compared with day 20—21 (*P* < 0.01; Fig. 4B).

### Experiment 3. The long-term effect of IUD on mRNA PG synthase transcription in mare endometrium in vivo

**Fig. 5:**
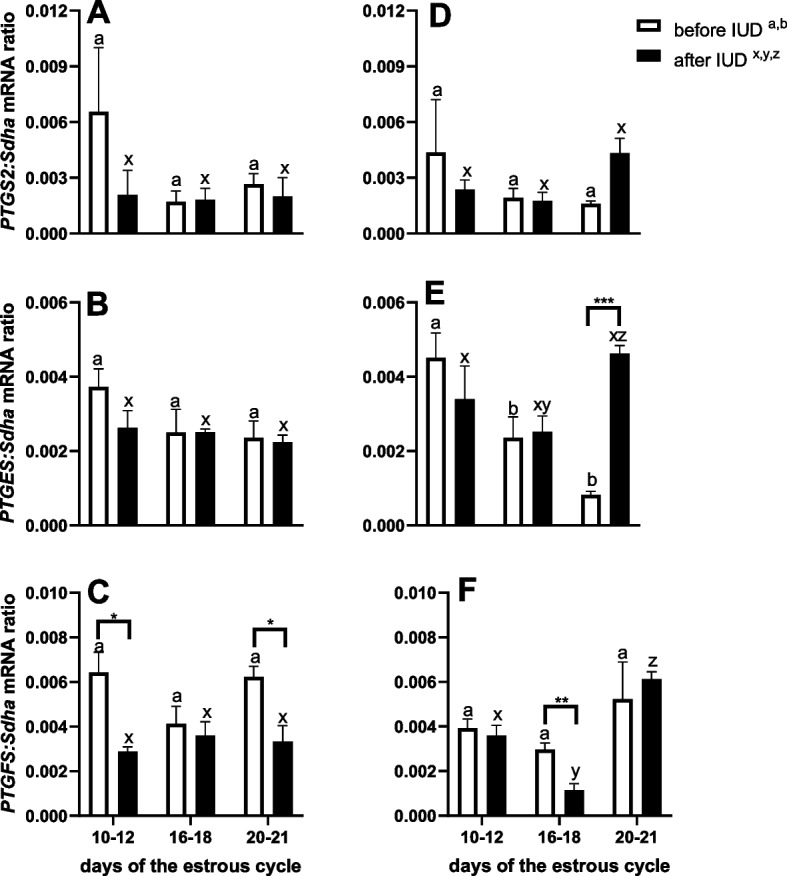
The long- term effect of an intrauterine device (IUD) inserted on day 0 (**A**,**B**, **C**) and IUD inserted on days 5–6 post-ovulation (**D**, **E**, **F**) on transcripts of prostaglandin-endoperoxide synthase-2 (*PTGS2*) (**A**, **D**), prostaglandin E_2_ synthases (*PTGES*) (**B**, **E**), and prostaglandin F_2α_ synthases (*PTGFS*) (**C**, **F**), in equine endometrial biopsies collected within the time intervals during the estrous cycle: days 10 -12, 16- 18, 20—21 from IUD mares compared with the control biopsies (collected on the same days of the estrous cycle before IUD insertion). Asterisks indicate significant differences (^*^P < 0.05; ^**^P < 0.01; ^***^P < 0.001) between endometrial biopsies collected before and after IUD insertion at the same time point. Superscript letters indicate significant differences between the control group (before IUD ^a,b^) across the time points (days of the estrous cycle) or between IUD mare group (after IUD.^x,y,z^) across the time points (days of the estrous cycle)

After IUD insertion on day 0, *PTGFS* mRNA transcription was lower in endometrial biopsies collected on days 10—12 and 20—21, compared with control biopsies collected on those same days of the estrous cycle before IUD insertion (*P* < 0.05; Fig. 5C). No difference was observed in *PTGS2* and *PTGES* mRNA transcription in endometrial biopsies collected at the specific time points (*P* > 0.05; Fig. 5A and B).

After IUD insertion on day 5 or 6, *PTGES* mRNA transcription increased in endometrial biopsies collected on day 20—21, compared with control biopsies collected on the same days of the estrous cycle before IUD insertion (*P* < 0.001; Fig. 5E). mRNA transcription of *PTGFS* was lower in endometrial biopsies collected on day 16—18 compared with control biopsies collected on those same days before IUD insertion (*P* < 0.01; Fig. 5F). No difference was observed in *PTGS2* mRNA transcription in endometrial biopsies collected at the specific time points (*P* > 0.05; Fig. 5D). After IUD insertion on day 5 or 6, *PTGES* mRNA transcription was higher in endometrial biopsies collected on day 20- 21 compared with biopsies collected on day 16 -to 18 (*P* < 0.001; Fig. 5E). Moreover, *PTGES* mRNA transcription was lower in control biopsies collected on days 16—18 and 20—21 compared with control biopsies collected on day 10- 12 (*P* < 0.001, Fig. 5E). *PTGFS* mRNA transcription was lower in endometrial biopsies collected on day 16—18 compared with biopsies collected on days 10—12 or 20—21 (*P* < 0.01; Fig. 5F).

No significant differences in mRNA transcription of *PTGS2*, *PTGES* and *PTGFS* between endometrial biopsies collected on day 20—21 of the estrous cycle from mares with a single (group 1), two (group 2) or three (group 3) endometrial biopsies were observed in the preliminary in vivo study (*P* > 0.05; Table 1).

**Table 1:** The effect of repeated sampling on the mRNA transcription of *PTGS2*, *PTGES*, or *PTGFS* in equine endometrium

	**Number sampling**		
	**3**	**2**	**1**
*PTGS2* mRNA transcription (mean ± SEM)	0.072 ± 0.018^a^	0.241 ± 0.059^a^	0.154 ± 0.057^a^
*PTGES* mRNA transcription (mean ± SEM)	0.159 ± 0.048^a^	0.244 ± 0.096^a^	0.047 ± 0.01^a^
*PTGFS* mRNA transcription (mean ± SEM)	0.352 ± 0.067^a^	0.993 ± 0.355^a^	0.34 ± 0.232^a^

Superscript letters ^a^ within the row indicate no significant between investigated groups. The results were considered significant when different at *P* < 0.05

Based on these results, we established that the number of biopsies taken had no effect on *PG synthase* mRNA transcription. The possible effect of the frequency of obtaining biopsies on *PG synthase* mRNA transcription was excluded. Therefore, in the in vivo Experiment 3, the biopsy samples were collected only once within the time intervals during the estrous cycle: days 10- 12, 16-18 and, 20-21.

## Discussion

Studies on equine MRP involving embryonic migration and early interactions between the conceptus and the uterine wall have recently focused on mechanical signaling, mechanotransduction, mechanoreceptors, and focal adhesion molecules [4, 21]. Previously, in vitro and in vivo studies examined the effect of embryo presence on the uterus and endometrial PG secretion [11, 14, 15, 22]. The recent studies concerning IUD use in mares have mainly focused on the IUD-induced effect on luteal lifespan [17, 18]. The present in vitro and in vivo studies provide information necessary for the understanding of the IUD mechanism to prolong luteal activity. We report the actions of IUD on the endometrial secretory function of PG showing that IUD acts as a potent modulator of PG secretion. We demonstrated (for the first time) that the incubation of the endometrial cells in the presence of IUD altered the pattern of *PG synthase* mRNA expression in both epithelial and stromal endometrial cells. It also appears that IUD modulates P_4_ production by the CL, allowing for a prolonged luteal phase in the mare. Importantly, we demonstrated that the endometrial response as PG release depends on specific time of IUD insertion. For this purpose, we compared the effect of IUD inserted on day 0 of the estrous cycle (oestrus) with the specific time when embryo reaches uterus after fertilization (day 5 to 6 post ovulation) in vivo. The mechanical interactions between the IUD and endometrial epithelium were investigated, which will then respond similarly as in conceptus – uterine wall interactions during embryonic migration.

Szóstek et al. [23] reported that epithelial cells of equine endometrium produce PG in response to ovarian steroids, which modulate reproductive processes such as implantation, cell proliferation, and angiogenesis. In the present in vitro study, mainly the epithelial cells reacted to culture with the presence of the IUD. This could be explained by the fact that in the endometrium the epithelial cells are in direct contact with the endometrial lumen and hence with the embryo. In our in vitro study, we showed that PG production by different types of endometrial cells cultured in the presence of the IUD on the shaking platform may be explained by an alteration/modulation of PG synthases transcription in epithelial and stromal cells. To the best of our knowledge, until now, this effect of endometrial cells incubated in the presence of IUD on the arachidonic acid (AA) metabolic pathway has not been studied. We demonstrated that the culture of the epithelial cells in the presence of the IUD resulted in a modified secretion of PGE_2_ and PGF_2α_ at 24 h. The endometrial cells incubated in the presence of IUD altered the mRNA transcription of *PTGES* and *PTGFS* synthases, consequently acting as a potent modulator of endometrial PG secretion.

In our in vitro study, the presence of the IUD diminished mRNA transcription of *PTGFS synthases* by epithelial cells, resulting in a decrease of PGF_2α_ secretion. Therefore, we assume that IUD mimicking the presence of an embryo in the endometrium is involved in the inhibition of luteolysis by attenuating the secretion of PGF_2α_. Previous in vitro studies focused mainly on the inhibitory effect of the presence of equine embryos on endometrial PG secretion. Sissener et al. [24] and Sharp et al. [25] demonstrated that in vitro incubation of day 14 endometrial explants with day 9 to 16 embryos or with embryonic membranes suppressed PGF_2α_. Berglund et al. [14] and Watson and Sertich [15] used endometrial explants to study incubation with embryos and reported that incubation of embryos with endometria from pregnant mares lowered PGF_2α_ concentrations in the medium. It has been shown that the presence of yolk sac membranes, and yolk sac and trophoblast, but not trophoblast alone downregulates the amount of PGF_2α_ release by the respective day 14 uterine tissue in vitro [14]. Additionally, the contact of the IUD with endometrial culture from biopsies of day 11 pregnant mares induced changes in the expression of focal adhesion molecules [4]. However, the only culture with an embryo alone was capable of decreasing PGF_2α_ secretion in endometrial biopsy samples from non-pregnant mares. The differences between our results and those of Klohonatz et al. [4] are mostly due to different experimental approaches used: endometrial cells versus biopsy endometrial tissues.

In a previous study, in vitro PGE_2_ production was unaffected by the presence of the embryo during the incubation with mare endometrial explants [15]. Tissue concentrations and in vitro release of PGE_2_ were similar in endometrial samples from pregnant and non-pregnant mares [15]. However, the present in vitro study focusing on the secretory activity of equine endometrial cells incubated with the presence of the IUD increased *PTGES* mRNA transcription by both epithelial and stromal cells, enhancing the secretion of PGE_2_ by both cells. Since that in mares, PGE_2_ plays a luteotropic role as an auto-paracrine factor stimulating P_4_ production by luteal steroidogenic cells in vitro [26], we suggest that the IUD-induced increase in PGE_2_ secretion may play a supporting role in P_4_ secretion during CL development.

It should be pointed out that until now, only the long-term effect of IUD on luteal activity has been studied. Riviera del Alamo et al. [17] found that in mares receiving an IUD on day 2 to 4 post ovulation, IUD induced a prolonged luteal phase in 75% of mares. Similarly, we showed that P_4_ concentrations were higher in blood samples collected from IUD groups of mares on days 16–18 and 20–21 compared with the control groups, suggesting prolonged luteal phase in examined mares.

In our in vivo study, we also determined whether the IUD effect on P_4_ appears immediately after IUD application (short-time effect of IUD). Thus, we conducted a frequent blood plasma collection during the first 24 h after IUD insertion on day 0 or day 5 or 6 post ovulation, respectively. After IUD insertion on day 5 to 6, P_4_ concentrations were temporally elevated 1 h after IUD insertion, whereas the P_4_ profile followed a pattern similar to that in the control group during 24 h after IUD insertion on day 0.

Blood plasma PGE_2_ concentrations increased after IUD insertion on day 0. To the best of our knowledge, this is the first in vivo study demonstrating the positive effect of IUD on the secretion of luteotropic PGE_2_ in mares. Previously, Lukasik et al. [26, 27] showed that PGE_2_ plays a luteotropic role as an auto-paracrine factor stimulating P_4_ production by luteal steroidogenic cells and CL tissues in vitro. We assume that the PGE_2_ rise observed in our study, is an indirect effect on equine PGE_2_ receptors in the uterus, affecting the regulation of vasculature events and induction of luteotropic factors involved in luteal support. However, further studies are needed to clarify the mechanism of action of IUD on PGE_2_ production within the equine reproductive tract. Riviera del Alamo et al. [17] examined the return of the luteolytic activity in mares inserted with an IUD by measuring PGFM profile in blood plasma collected between days 11 to 16 of the estrous cycle, suggesting that the contact of the IUD with endometrium may prevent endometrial cells from releasing PGF_2α_ [17]. In our in vivo study, the IUD did not affect blood plasma PGFM concentration within 24 h of the experiment, independently of the time of the IUD insertion. Therefore, we suggest that prolonged physical contact of the IUD and its interactions with the inner uterine wall are necessary to affect CL function in mares.

A recent study conducted by NewCombe et al. [28] has reported that the timing of the MRP signal varies for each mare. Moreover, it has been observed that the delivery of the MRP signal is repeatable in mares within a post-ovulation window of 24 h or less for each animal. In the mare conceptus mobility plays an important role in the MRP [29]. However, the reason why the conceptus needs to be mobile is still not fully understood. It could be due to the mechanical interaction between the embryo and the endometrium, the chemical instability of the MRP signal, or the necessity for the embryo to receive enough nourishment through the secretions of the endometrial glands to ensure its survival [21]. Nevertheless, the necessity of embryo mobility is questioned by some researchers. Previously, Wilsher et al. [30] observed that in some mares receiving transferred embryos, embryo movement stopped on day 9, yet luteostasis still occurred. More recently, Wilsher et al. [31] demonstrated that manual reduction of day 11 embryos 24 or 12 h after transfer to recipient mares on days 10, 11, 12 or 13 post-ovulation resulted in 93% entering a period of luteostasis. In the another study of Wilsher et al. [32], it was observed that transferering day 11 ruptured embryos can prevent luteolysis in recipient mares. For proper implantation of the embryo, the endometrial surface requires a signal from MRP. However it is still not clear whether the MRP signal is a single chemical signal released by the conceptus, a mechanical signalling, or consists of multiple pathways, as suggested by Sharp et al. [33] and Swegen [21]. During this period, there are many unknown factors, and intensive research is necessary to explain MRP in mare due to its multifactorial nature. Therefore, the role of mechanoreceptors located in equine endometrium and myometrium should be also considered [21]. The role of mechanotransduction is well established in different tissues (e.g. bone tissue), which indicates interactions between mechanical stimuli and PTGS2 expression, whereby PGE_2_ synthesis is increased in response to mechanical forces via the formation of local adhesions [34]. Thus, the IUD or the embryo could trigger a mechanoreceptor response that modulates PTGS2 activity and affects PG production in the endometrium [21]. Taking into account that release of PG was affected by the IUD insertion in our in vivo study, we decided to examine the transcription pattern of the enzymes involved in PG biosynthesis. Previously, Riviera del Alamo et al. [18] found that application of IUD inhibited the upregulation of PTGS2 protein abundance on day 15 that disrupts the luteolytic pathway. They put forward the hypothesis that phospholipase 2, required for releasing AA from membrane cells, could initially be blocked by IUD. In our in vivo study, no effect of IUD insertion on day 0 or days 5 or 6 on *PTGS2* mRNA transcription was found in the endometrial biopsies collected on the chosen days of the estrous cycle. More importantly, we have noticed a decrease in *PTGFS* mRNA transcription in endometrial biopsies collected on days 10—12 and 20—21 after the insertion of IUD on day 0. Furthermore, *PTGFS* mRNA transcription was downregulated in endometrial biopsies collected on day 16- 18 in mares with IUD inserted on days 5 or 6. Moreover, in this group of mares, we have observed an increase in *PTGES* mRNA transcription in biopsy samples collected on day 20- 21. The decrease of *PTGFS* mRNA transcription associated with an elevation in *PTGES* mRNA transcription in biopsies may suggest an antiluteolytic action of IUD during the estrous cycle. We propose that the mechanism by which the IUD induces luteal phase prolongation is by modifying the expression of the enzymes involved in the synthesis of PG.

## Conclusion

In conclusion, the application of IUD may mimic the equine conceptus presence through the physical contact with the endometrium altering transcription of PG synthases, thus acting as a potent modulator of endometrial PG secretion both in vitro and in vivo. The blood plasma PG levels or *PG synthase* mRNA transcription in the endometrium are different depending on day of the IUD insertion in the mare (day 0 vs day 5–6 of the estrous cycle). The decrease of *PTGFS* mRNA transcription on day 16- 18, associated with an elevation in *PTGES* mRNA transcription on day 20 21 in endometrial biopsies in mares placement with IUD on day 5–6 suggest an antiluteolytic action of IUD during the estrous cycle. The upregulation of *PTGES* and downregulation of *PTGFS* mRNA transcription in epithelial cells, and upregulation of *PTGFS* mRNA transcription in stromal cells seem to be the main endometrial cell responses to incubation with the IUD (on PG secretion). The migration of the equine embryo in the uterine lumen may affect PG production, not only by secreted molecules but also by physical contact. The effect of IUD on in vivo endometrial PG secretion and on endometrial cells incubated with the presence of IUD confirm the importance of the physical contact of IUD with the endometrium on the extension of CL lifespan and the luteotropic function in mares.

## Methods

### Intrauterine device preparation for in vitro and in vivo study

The intrauterine device – a polypropylene ball filled with phosphate-buffered saline (PBS) with a diameter of 20 mm and an average weight of 3.6 g was sterilized by boiling in water for 10 min as previously described by Rivera del Alamo et al. [17]. The IUD prepared this way was used in the present in vitro study. Moreover, IUD was inserted through the cervix into the mare’s uterus in our in vivo study.

### Collection of mare endometrial tissues

Uteri (*n* = 10) at the early luteal phase were collected post mortem from cyclic draught mares aged from 3 to 5 years, weighing between 500 and 700 kg, at a commercial abattoir (Rawicz, Poland). The animals were raised on a farm as food animals and slaughtered for meat. The mares had been declared clinically healthy by the official government veterinary inspection and by individual health records. The uteri were obtained over the reproductive season (between April and July). Samples of peripheral blood were collected from the jugular vein into heparinized tubes immediately before slaughter for P_4_ analysis. The determination of the phase of the cycle was based on analysis of P_4_, as well as on the macroscopic observation of the ovaries [35]. The early luteal phase was characterized by the presence of a corpus hemorrhagicum with a plasma concentration of P_4_ > 1 ng/ml.

For the histological analysis, a small piece of endometrial tissue (1 × 1 cm) from each uterus was placed in 4% buffered paraformaldehyde for hematoxylin–eosin staining [35], and further grading according to Kenney and Doig´s classification system [36]. Only cells derived from category I endometria were used in the present study.

For epithelial and stromal cell isolation, the entire uterus was collected within 5 min of animal’s death, and placed in sterile, incomplete (Ca2 +—and Mg2 +—free) Hanks’ balanced salt solution (HBSS) supplemented with gentamicin (20 μg/ml; Sigma-Aldrich, Madison, USA, #G1272) and 0.1% bovine serum albumin (BSA; Sigma–Aldrich, Madison, USA, #A9056), kept on ice, and transported to the laboratory.

### In vitro study design

#### Experiment 1. The time-dependent effect of IUD on PG in endometrial cells in vitro

**Fig. 6:**
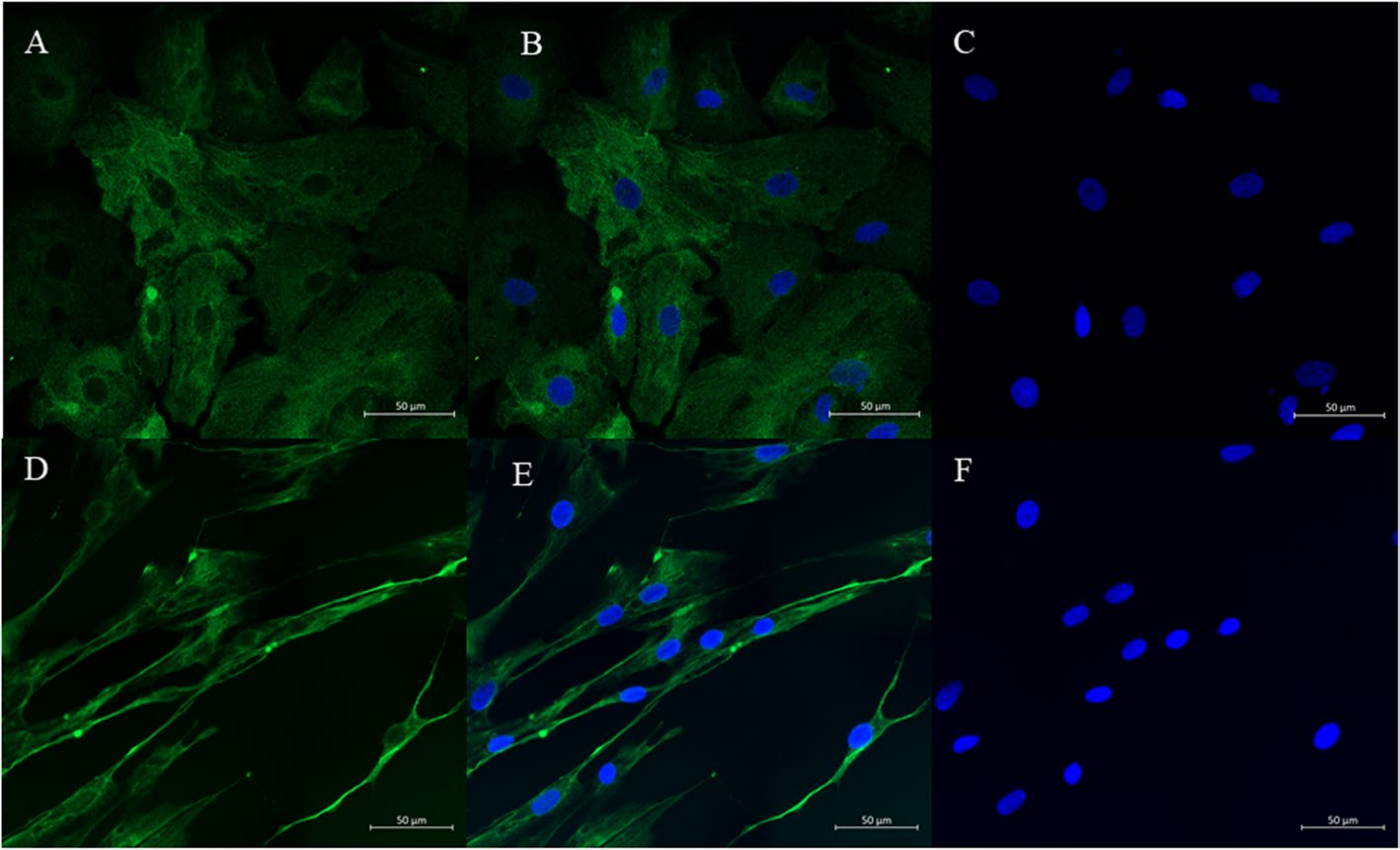
Representative pictures of immunofluorescence staining of cytokeratin (**A**, **B**) and vimentin (**D**, **E**) in cultured epithelial and stromal cells in the mare endometrium. The scale bar = 20 μm (magnification: 40) **C**, **F**-DAPI staining

The effects of IUD on PGE_2_ and PGF_2α_ production and the expression of genes involved in PG synthesis was investigated in mare endometrial cells in vitro. The epithelial and stromal cells were isolated following the methodology previously described [37]. Cell culture homogeneity was evaluated using immunofluorescent staining for epithelial and stromal cell specific markers (cytokeratin and vimentin respectively) as described previously [38]. Representative pictures of immunofluorescence staining of cytokeratin and vimentin in cultured epithelial and stromal cells is shown in Fig. 6. The cells were seeded at a density of 1 X 10^5^ viable cells/ml for epithelial cells and 2.5 X 10^5^ viable cells/ml for stromal cells. The viability of both cell types was over 92%. Epithelial (*n* = 5 for each time point) and stromal cells (*n* = 5 for each time point) derived from passage I were placed in a 6–well plate in culture medium DMEM/Ham’s F–12 supplemented with 10% FCS and 1% antibiotic antimycotic solution. When the cells reached confluence, the medium was replaced with fresh DMEM without phenol red (Sigma–Aldrich, Madison, USA; #D2906) supplemented with gentamycin (20 μg/ml) and 0.1% BSA. Then, the epithelial and stromal cells were incubated in the presence of PBS–filled ball (one IUD per well) or without IUD (control group) on a shaker inside the incubator for 6, 12, and 24 h. The plate was gently shaken (shaking speed 50 rpm) during the experiment to mimic the embryo presence in the uterus. To make sure that IUD did not cause damage in culture, the cells were observed at each time point of the incubation. No cell detachment was detected after their incubation with the presence of IUD.

**Fig. 7:**
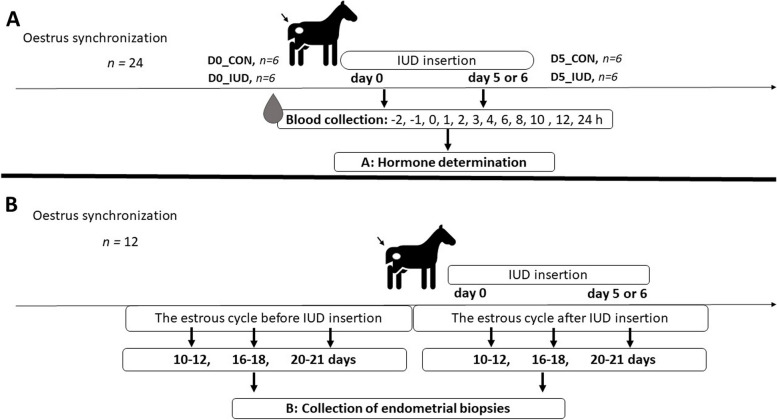
Graphical representation of the in vitro experimental design. IUD = intrauterine device

At 6, 12, and 24 h after culture of the cells in presence of IUD, the culture medium was collected in tubes containing 5% ethylenediaminetetraacetic acid (EDTA) and 1% acetylsalicylic acid solution (Sigma–Aldrich, #A2093, pH = 7.4). Samples were kept frozen at –20°C until PGE_2_ and PGF_2α_ concentrations were determined by ELISA. For single–step DNA isolation, 250 µl of TRI Reagent (Sigma-Aldrich, Madison, USA, #T942) was added to each well containing cells. Cells were then collected from wells. Deoxyribonucleic acid was isolated according to TRI Reagent manufacturer`s procedure. DNA content was used to standardize results. At 24 h after co-culture of the cells with IUD, the culture medium was removed and 250 µl of fenozol (A&A Biotechnology, Gdansk, Poland) was added into each well for PG synthases transcription assays using qPCR. A graphical representation of the in vitro experimental design is depicted in Fig. 7.

### Animals and treatments

The in vivo study was carried out on draught mares (*n* = 36) aged from 3 to 5 years, weighing from 500 to 700 kg. The animals had normal estrous cycles throughout the study period (between June and August). All were clinically healthy with no history of reproductive abnormalities. The mares were housed in a private stable in boxes, fed dry hay and commercial concentrate mix with free access to water and trace mineral salt blocks. All mares were routinely subjected to regular management procedures, such as deworming and vaccinations.

Before the experiment, estrous cycles of the mares were synchronized using a PGF_2α_ analogue (5 mg/ ml dinoprost, Dinolytic, Zoetis, Poland) according to the manufacturer’s instructions. Mare’s genital tract was examined every day by ultrasonography with a 7.5 MHz linear probe (MyLabOne Vet Ultrasound System, ESOATE Pie Medica, Genoa, Italy) for detection of follicles, ovulation (day 0), follicle size, and the degree of endometrial oedema. Moreover, a clinical exam including physical examination, heart rate, and rectal temperature was performed to ensure that the horses were healthy.

### Blood sampling collection

Mares were sedated with detomidine hydrochloride (Domosedan 0.01 mg/kg iv, Orion Pharma Poland Sp, Poland) followed by the insertion of a temporary catheter (Intraflon IV cannula 2.1 X 80 mm 14G; KRUUSE, 121,805, KRUUSE Poland) into the jugular vein. Intravenous catheters were flushed with heparinized saline and used for frequent blood sample collections. Blood was aspirated into sterile 10 ml tubes containing 100 μl of 0.3 M EDTA (POCH, #879810112) and 1% acetylsalicylic acid, pH 7.4 (Sigma-Aldrich, Madison, USA, #A2093). After centrifugation (2 000 X g, 10 min at 4 °C), the plasma was stored at − 20 °C for determination of P_4_, PGE_2_, and 13, 14- dihydro-15-keto PGF_2α_ (PGFM) concentrations by RIA or ELISA, respectively.

### Collection of mare endometrial biopsies

Endometrial biopsies were collected by the same operator according to the previously described method [39]. One biopsy per mare was obtained from the ventral wall at the base of the uterine horn for each time-point during the estrous cycle before and after IUD insertion. Biopsies weighed from 320 to 450 mg each. Biopsy samples were divided with sterile scissors and (i) immersed in 1 ml of RNALater (Sigma-Aldrich Steinheim, Germany, #R0901) and stored at –80 °C until RNA extraction or (ii) placed in 4% buffered paraformaldehyde (POCH, Gliwice, Poland, # #432173111) for histological analysis, as described previously [35]. All endometria were classified according to the classification system of Kenney and Doig [36]. Only endometrial tissues graded as category I were used in the study.

### In vivo studies design

#### Experiment 2. The short-term effect of the IUD inserted on day 0 versus days 5–6 of the estrous cycle on blood hormonal profile in mare in vivo

**Fig. 8:**
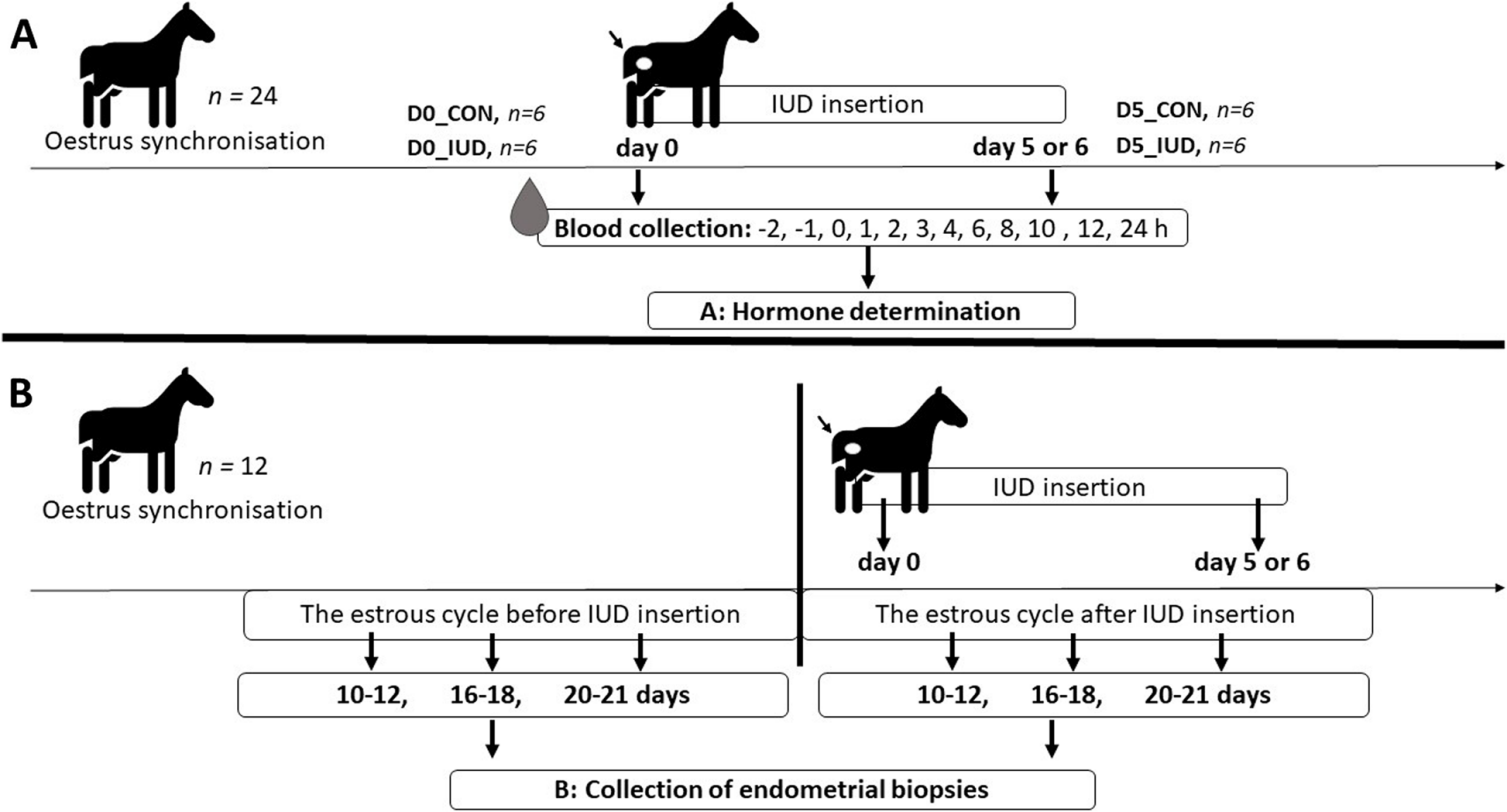
Graphical representation of the in vivo experimental design. **A** experiment 2, **B** experiment 3. IUD = intrauterine device

This experiment aimed to compare short-time effect of IUD inserted on days 0 (oestrus) with 5–6 post -ovulation (specific time when embryo reaches uterus after fertilization) on blood plasma PG concentrations in mares. For a better explanation of the different experiments carried out in the present in vivo study, a graphical representation of the experimental design is depicted in Fig. 8.

The mares (*n* = 24) were used in the in vivo experiment 1 study (Fig. 8A). In the group of mares placement with IUD on day 0 of the estrous cycle, the animals were divided into: (i) control mares (D0_CON; *n* = 6), and (ii) IUD mares (D0_IUD; *n* = 6). In the group of mares placement with IUD on day 5 or 6 of the estrous cycle, the animals were divided into: (iii) control mares (D5_CON; *n* = 6), and (iv) IUD mares (D5_IUD; *n* = 6). The control mares were exposed to the same manipulation but without IUD (cervical manipulation). The catheter was placed 2 h before the placement of IUD, followed by sampling at − 2, − 1, 0, 1, 2, 3, 4, 6, 8, 10, 12, and 24 h after IUD insertion. The time of IUD insertion was defined as 0 h. Concentrations of P_4_, PGE_2_, and PGFM were measured in blood plasma.

Additionally, to determine the effect of IUD on luteal phase in mares, a single blood sample for P_4_ determinations was collected only once within the time intervals during the estrous cycle: days 10–12, 16–18, and 20–21. The experimental groups were identified as day 10–12, day 16–18, or day 20–21 due to possible variation in the time of ovulation (± 1 day) in mares.

#### Experiment 3. The long-term effect of IUD on mRNA PG Synthases transcription in mare endometrium in vivo

This experiment aimed to compare the long-term effect of IUD inserted on days 0 (oestrus) with 5–6 post ovulation (specific time when embryo reaches uterus after fertilization) on PG synthase transcript in endometrial biopsies collected from mares. For a better explanation of the different time points when endometrial biopsies were collected in the present in vivo study, a graphical representation of the experimental design is depicted in Fig. 8B. The mares (*n* = 12) were placement with IUD on following days of the estrous cycle: (i) day 0 (*n* = 6), and (ii) day 5 or 6 (*n* = 6), respectively. Endometrial biopsies were taken only once within the time intervals during the estrous cycle: days 10–12, 16–18, and 20–21. Biopsies samples taken during the estrous cycle before IUD insertion were determined as the internal control for each mare. The experimental groups were identified as day 10–12, day 16–18, or day 20–21, due to possible variation in the time of ovulation (± 1 day) in mares.

To establish if biopsy frequency may directly impact on *PG synthase* mRNA transcription in equine endometrium the preliminary in vivo study was performed (Table 1).Mares were assigned to three groups: (i) Group 1 (3 biopsies, *n* = 4) mares with endometrial biopsies collected only once within the time intervals during the estrous cycle: days 10–12, 16–18, and 20–21; (ii) Group 2 (2 biopsies, *n* = 4) mares with endometrial biopsies collected only once within the time intervals during the estrous cycle: days 16- 18, and 20 21; and, (iii) Group 3 (1 single biopsy, *n* = 4) mares with endometrial biopsies collected only once within the time interval during the estrous cycle: day 20–21 Only endometrial biopsies collected form mares (Group 1, 2, 3) during the time interval—day 20–21 were used to examine the effect of multiple biopsies on mRNA *PG synthases* transcription in the endometrium.

### Analytic methods

#### Total RNA extraction and cDNA synthesis

Total RNA was extracted from endometrial biopsy samples and cultured from equine endometrial cells using the Total RNA Mini kits (A&A Biotechnology, Gdansk, Poland, #031–100), according to the manufacturer’s instructions. Content and purity of RNA were assessed using a NanoDrop 1000 Spectrophotometer (Thermo Fisher Scientific, ND-1000, Wilmington, DE, USA). The A260/280 absorbance ratio for all samples was approximately 2.0, and the 260/230 absorbance ratio ranged between 1.8 and 2.2. Then, 1 μg RNA was reverse-transcribed into cDNA using a Reverse Transcription Kit (Qiagen, Hilden, Germany, #205311) according to the manufacturer’s instructions.

#### Quantitative real-time PCR

Quantitative real-time PCR (qPCR) was performed in 384-well plates with an ABI 7900 HT system (Applied Biosystems, Foster City, CA, USA) using SYBR Green PCR master mix (Applied Biosystems, Foster City, CA). The sequences used for equine PTGS2, PTGFS and PTGES primers have been published by Szóstek et al. [40]. All primers were synthesized by Sigma (Custom Oligos Sigma-Aldrich, Haverhill, United Kingdom). The validation of the reference gene was carried out using NormFinder program. *Succinate dehydrogenase complex subunit A* (*Sdha*), *β-actin* (*ACTB*), *2β-microglobulin* (*2BM*), *glyceraldehyde-3-phosphate dehydrogenase* (*GAPDH*), *hypoxanthine phosphoribosyltransferase* (*HPRT*) were tested for stability as reference genes. *Succinate dehydrogenase complex, subunit A* (*Sdha*) were most stable among tested genes and was chosen as the reference gene as reported previously [38]. The total reaction volume was 10 µl: 3 µl cDNA (10 ng), 1 µl forward and reverse primers each (500 nM) and 5 µl SYBR Green PCR master mix. qPCR protocol consisted of an initial denaturation (10 min at 95 °C), followed by 40 reaction cycles that included denaturation (15 s at 95 °C) and annealing (1 min at 60 °C). After each qPCR reaction, melting curves were obtained by stepwise increases in temperature from 60 °C to 95 °C to ensure single-product amplification. Control reactions without template or primers were performed to confirm that products were free from primer-dimers and genomic DNA contamination, respectively. qPCR results were analysed using the method described by Zhao and Fernald [41], which based on the DeltaCT analysis. It is objective method for quantifying qPCR results using calculations based on the kinetics of individual PCR reactions without the need of the standard curve, independent of any assumptions or subjective judgments which allow direct calculation of efficiency and CT. The relative concentration of mRNA (R0) for each target and reference gene (*Sdha*) was calculated using the equation R0 = 1/(1 + E)Ct, where, E is the average gene efficiency and Ct is the cycle number at the threshold. The relative gene expression was calculated as R0target gene/R0reference gene and was expressed in arbitrary units.

#### Hormone determinations

Prostaglandin F_2α_ concentration in conditioned media was determined using a commercial ELISA kit (Enzo Life Science, # ADI-901–069) according to the manufacturer’s instructions, as previously described [42–44]. The standard curve for PGF_2α_ ranged from 3 pg/ml to 50 000 pg/ml. The sensitivity of the PGF_2α_ assay was 6.71 pg/ml. The intra- and inter-assay CVs were 9 and 11%, respectively.

Prostaglandin E_2_ concentration in conditioned media or blood plasma was determined using a commercial ELISA kit (Enzo Life Science, Farmingdale, New York, USA, #ADI-901–001) according to the manufacturer’s instructions, as previously described [42–44]. The standard curve for PGE_2_ ranged from 19.3 to 2 500 pg/ml. The sensitivity of the PGE_2_ assay was 13.4 pg/ml. The intra- and inter-assay CVs were 9% and 12.8%, respectively.

Progesterone concentration in blood plasma was measured using P_4_ 125 104 I RIA kit (Immunotech, Czech Republic, #IM1188) according to the manufacturer’s instructions. The standard curve for P_4_ ranged from 0.1 to 100 ng/ml. The intra- and inter-assay CVs were 6.5% and 8.1%, respectively.

13, 14- dihydro-15-keto PGF_2α_ (PGFM) concentration in blood plasma was determined using a commercial ELISA kit (Cayman Chemical Company, Ann Arbor, Michigan, USA, No. 516671) according to the manufacturer’s instructions, as previously described [45, 46]. The standard curve for PGFM ranged from 2.3 to 5 000 pg/ml. The sensitivity of the PGFM assay was 80% B/B0:15 pg/ml. The intra- and inter-assay CVs were 8.8% and 6.8%, respectively.

### Statistical analysis

Prior to each analysis, the Gaussian distribution of results was tested using the Shapiro–Wilk test or the D’Agostino & Pearson normality test (GraphPad). Whenever the assumptions of normal distribution were not met, nonparametric statistical analyses were done.

In the in vitro Experiment 1 statistical analysis of the time–dependent effect of IUD on PG secretion in endometrial cells was performed using Multiple t test with Mann–Whitney test (GraphPad Software version 8.3.0, GraphPad Software, San Diego, CA, USA). Statistical analysis of *PG synthases* mRNA transcription was performed using nonparametric Mann–Whitney U test (GraphPad). In the in vivo Experiments 2 and 3 statistical analyses were performed using two-way ANOVA test followed by the Sidak’s multiple comparison test (GraphPad). In the preliminary in vivo study statistical analysis was performed using a one-way ANOVA test followed by Tukey’s multiple comparison test (GraphPad). Results were considered significantly different at *P* < 0.05.

## Data Availability

The datasets used and analysed during the current study are available from the corresponding author on reasonable request.

## Abbreviations

ANOVA: Analysis of variance
BSA: Bovine serum albumin
CL: Corpus luteum
IUD: Intrauterine device
P_4_: Progesterone
PBS: Phosphate-buffered saline
PCR: Polymerase chain reaction
PGE_2_: Prostaglandin E2
PGF_2α_: Prostaglandin F2α
PTGS2: Prostaglandin-endoperoxide synthase 2
PGES: Prostaglandin E_2_ synthase
PGFS: Prostaglandin F_2α_ synthase
